# IPA1 functions as a downstream transcription factor repressed by D53 in strigolactone signaling in rice

**DOI:** 10.1038/cr.2017.102

**Published:** 2017-08-15

**Authors:** Xiaoguang Song, Zefu Lu, Hong Yu, Gaoneng Shao, Jinsong Xiong, Xiangbing Meng, Yanhui Jing, Guifu Liu, Guosheng Xiong, Jingbo Duan, Xue-Feng Yao, Chun-Ming Liu, Hongqing Li, Yonghong Wang, Jiayang Li

**Affiliations:** 1 State Key Laboratory of Plant Genomics and National Center for Plant Gene Research (Beijing), Institute of Genetics and Developmental Biology, Chinese Academy of Sciences, Beijing 100101, China; 2 University of Chinese Academy of Sciences, Beijing 100049, China; 3 Key Laboratory of Plant Molecular Physiology, Institute of Botany, Chinese Academy of Sciences, Beijing 100093, China; 4 Guangdong Provincial Key Lab of Biotechnology for Plant Development, South China Normal University, Guangzhou, Guangdong 510631, China; 5 Present address: Department of Genetics, University of Georgia, Athens, Georgia 30605, USA; 6 Present address: College of Horticulture, Nanjing Agricultural University, Nanjing, Jiangsu 210095, China; 7 Present address: Agricultural Genome Institute at Shenzhen, China Academy of Agricultural Science, Shenzhen, Guangdong 518120, China

**Keywords:** tillering, plant architecture, phytohormone, protein interaction, *Oryza Sativa* L.

## Abstract

Strigolactones (SLs), a group of carotenoid derived terpenoid lactones, are root-to-shoot phytohormones suppressing shoot branching by inhibiting the outgrowth of axillary buds. DWARF 53 (D53), the key repressor of the SL signaling pathway, is speculated to regulate the downstream transcriptional network of the SL response. However, no downstream transcription factor targeted by D53 has yet been reported. Here we report that *Ideal* *Plant* *Architecture* *1* (*IPA1*), a key regulator of the plant architecture in rice, functions as a direct downstream component of D53 in regulating tiller number and SL-induced gene expression. We showed that D53 interacts with IPA1 *in vivo* and *in vitro* and suppresses the transcriptional activation activity of IPA1. We further showed that IPA1 could directly bind to the *D53* promoter and plays a critical role in the feedback regulation of SL-induced *D53* expression. These findings reveal that IPA1 is likely one of the long-speculated transcription factors that act with D53 to mediate the SL-regulated tiller development in rice.

## Introduction

Strigolactones (SLs), a collection of terpenoid-derived compounds produced by plants, were firstly identified as the host-derived germination signals of root parasitic plants^1^, and later as stimulants of hyphal branching of arbuscular mycorrhizal fungi^2^. The characterizion of *more axillary growth* (*max*) mutants in *Arabidopsis thaliana*^3,4,5,6,7,8,9^, *dwarf* (*d*) mutants in rice (*Oryza Sativa*L.)^10,11,12,13,14,15,16^, *ramosus* (*rms*) mutants in pea (*Pisum sativum*)^4,17^, and *decreased apical dominance* (*dad*) mutants in petunia (*Petunia hybrida*)^18,19,20^ have indicated that SLs function as root-to-shoot phytohormones that regulate shoot branching^21,22^ and also regulate leaf senescence and root development^23,24,25,26,27,28^. Subsequently, the elucidation of the biosynthetic and signaling pathways of SLs has attracted great attention. Carlactone is the conserved endogenous precursor of SLs, which is derived from all-*trans*-β-carotene through DWARF27 (D27)-mediated isomerization and subsequent cleavage by the carotenoid cleavage dioxygenases 7 and 8 (CCD7 and CCD8)^14,29,30^. In *Arabidopsis thaliana*, cytochrome P450 MAX1 has been shown to oxidize carlactone to carlactonoic acid (CLA)^31^, which is then converted into methyl carlactonoate (MeCLA) and further catalyzed to an unidentified strigolactone-like compound by an oxidoreductase-like enzyme, Lateral Branching Oxidoreductase (LBO)^32^. However, in rice, carlactone is first converted into *ent*-*2*′-*epi*-*5*-*deoxy*-strigol by one MAX1 homolog and further into orobanchol by a second MAX1 homolog^33^. Highly branched phenotypes that can be rescued by SL treatment are displayed by SL biosynthesis deficient mutants that include *d27*, *d10*, and *d17* in rice; *rms1* in pea; and *max1*, *max3*, *max4*, and *lbo* in *Arabidopsis thaliana*^14,21,22,31,32^.

Compared with the SL biosynthetic pathways, the knowledge of SL-mediated signaling is still obscure. Through studying SL insensitive mutants in rice, three components have been identified involved in SL perception and signaling. *DWARF14* (*D14*) encodes a member of the α/β-hydrolase fold family protein, which binds and hydrolyses SL to form a covalently linked intermediate molecule (CLIM)^11,12,15,34,35,36,37^. This reaction triggers a conformational change to form a complex with DWARF3 (D3), which encodes an F-box protein^13,38,39^. D14 could interact with D3 and DWARF53 (D53) in the presence of SLs, leading to the ubiquitination and degradation of the nuclear-localized repressor D53^38,39^. D53 contains three ethylene-responsive element binding factor-associated amphiphilic repression (EAR) motifs, which are essential to recruit the transcriptional co-repressor TOPLESS (TPL) proteins and induce their oligomerization to form a repressor-corepressor-nucleosome complex^40^. In accord, D53 was shown to interact with a TPL-related protein, TPR2, in rice^38^. However, whether D53 regulates the transcription of genes in the endogenous SL signaling pathway and what are its direct downstream transcription factors remain to be determined. In other angiosperms such as *Arabidopsis thaliana* and pea, the orthologs of *D14*, *D3* and *D53* have similar functions^6,7,9,41,42,43,44^.

*Ideal Plant Architecture1* (*IPA1*) is a key regulator in determining plant architecture, which encodes a member of the SQUAMOSA PROMOTER BINDING PROTEIN-LIKE (SPL) family transcription factors, SPL14^45,46^. Two microRNAs, miRNA156 and miRNA529, regulate *IPA1* expression, and point mutations in the miRNA156 recognition site perturb miRNA156-regulated degradation of *IPA1* mRNA, leading to decreased tiller number and increased plant height and panicle branches^45,46,47^. In contrast, the CRISPR/Cas9-generated *ipa1* loss-of-function mutants exhibited opposite phenotypes^48^, reminiscent of the dwarf and high tillering phenotype of SL-deficient or -signaling mutant plants. IPA1 directly binds to the promoter of a negative regulator of tiller bud outgrowth, *TEOSINTE BRANCHED1* (*OsTB1)*, to suppress rice tillering^47^. The ortholog of *OsTB1* in *Arabidopsis thaliana*, *BRANCHED1* (*BRC1*), is a key regulator of branch outgrowth and one of the SL-responsive genes^49,50^. Previous studies showed that SL-induced bud growth inhibition occurs at least partially through the regulation of *BRC1* transcription in *Arabidopsis thaliana* and pea^41,51,52,53^. These results lead to the hypothesis that D53 sequesters transcriptional co-repressor TPL proteins to suppress IPA1, a key transcription factor regulating tillering. In this study, we reported that IPA1 is the direct downstream component targeted by D53 to regulate the SL response and SL-induced gene expression in rice. D53 interacts physically with IPA1 and inhibits its transcriptional activation activity. Furthermore, IPA1 binds directly to the promoter of *D53* and plays a critical role in the feedback regulation of SL-induced *D53* expression. Together, these findings provide new insights into understanding the function of D53, and identify IPA1 as one of the D53-targeted downstream transcription factors in the SL signaling pathway in rice.

## Results

### Generation and phenotypes of *IPA1* loss-of-function mutants

**Figure 1:**
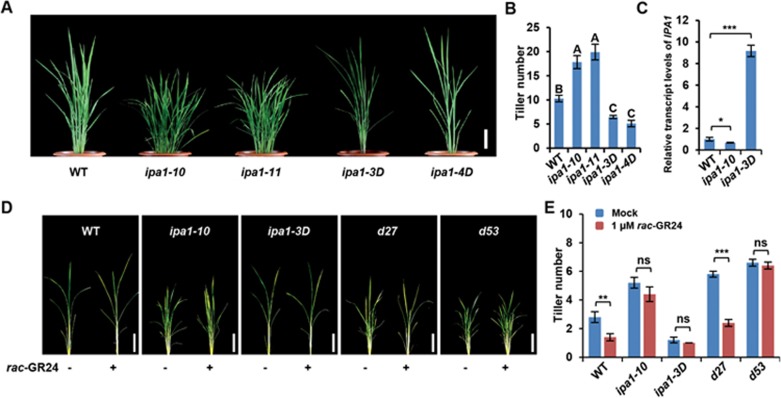
Phenotypic characterization of *IPA1* loss-of-function mutants. **(A)** Gross morphologies of wild-type (WT), *ipa1-10*, *ipa1-11*, *ipa1-3D*, and *ipa1-4D* plants before the heading stage. Bar = 10 cm. **(B)** Statistical analysis of tiller number in **(A)**. Values are means ± sem (*n* = 11). Different letters at top of each column indicate a significant difference at *P* < 0.05 determined by Tukey's HSD test. **(C)** *IPA1* expression levels in WT, *ipa1-10*, and *ipa1-3D.* Values are means ± SD (*n* = 3). The asterisks represent significant differences determined by Student's *t* test. ^*^P < 0.05, ^**^P < 0.001. **(D)** Gross morphologies of wild-type (WT), *ipa1-10*, *ipa1-3D*, *d27*, and *d53* seedlings with or without *rac*-GR24 treatment. Seedlings were treated with 1.0 μM *rac*-GR24 (+) or mock (–). Bar = 5 cm. **(E)** Statistical analysis of tiller number in **(D)**. Values are means ± SD (*n* = 5). The asterisks represent significant differences determined by Student's *t* test. ^**^P < 0.01, ^**^P < 0.001. ns, no significant difference.

Our previous study has shown that *IPA1* functions as a transcriptional activator in regulating tiller number and that its expression is regulated at both mRNA and protein levels; mRNA-level regulation was demonstrated by isolating and characterizing the two gain-of-function mutants, *ipa1-1D* and *ipa1-2D*^45,54,55^. To understand whether loss-of-function mutations of *IPA1* could affect rice plant architecture, especially tillering, we used a targeting induced local lesions in genomes (TILLING) method to generate point mutations in *IPA1*, which led to amino acid substitutions in the gene product. However, none of the mutant lines exhibited obvious phenotypes, suggesting that these amino acids are not essential for the function of IPA1 (Supplementary information, Figure S1). By applying a genome editing approach, four lines of *ipa1* mutants were generated, including two loss-of-function mutants, *ipa1-10* and *ipa1-11*, and two gain-of-function mutants, *ipa1-3D* and *ipa1-4D*^48^. The *ipa1-10* mutant resulted from a 5-bp deletion in the coding sequence; the *ipa1-11* mutant arose from a 57-bp insertion in the replacement of 102-bp deletion in the coding sequence; *ipa1-3D* and *ipa1-4D* were gain-of-function mutants resulting from 12-bp and 21-bp in-frame deletions in the miRNA156/529 target sites respectively (Supplementary information, Figure S2), which abolish the miRNA regulation without interfering the normal function of IPA1. The transcript and phenotypic analyses showed that the *ipa1-10* and *ipa1-11* loss-of-function mutants exhibited high tillering and dwarf phenotypes (Figure 1A-1C), indicating that IPA1 is a negative regulator of rice tillering. In contrast, both *ipa1-3D* and *ipa1-4D* homozygous plants have high levels of *IPA1* mRNA accumulation and low tillering (Figure 1A-1C).

Based on previous findings that the phenotypes of several rice tillering mutants are subject to SL regulation^14,22,38^, we therefore investigated whether IPA1 functions in the SL signaling pathway by examining the SL sensitivity of the respective loss- and gain-of-function mutants, *ipa1-10* and *ipa1-3D*, in response to *rac*-GR24, a synthetic SL analogue. The mutant *d27* showed a high tillering phenotype, due to a SL biosynthesis defect, which could be rescued by exogenously applied SL^14^. In the *d53* plant, the gain-of-function mutant D53 protein is resistant to ubiquitination and degradation. This blocks SL signaling and results in dwarf plants with high tillering phenotypes, which could not be rescued by exogenously applied SL^38,39^. Hence *d27* and *d53* mutants were used as the respective, negative and positive controls in this assay. Upon SL treatment, tiller number is unchanged in *ipa1-10* and *ipa1-3D* mutants, suggesting that they are insensitive to SL (Figure 1D and 1E). These results demonstrate that IPA1 plays an important role in the SL signaling pathway.

### IPA1 interacts with D53 protein *in vivo* and *in vitro*

**Figure 2:**
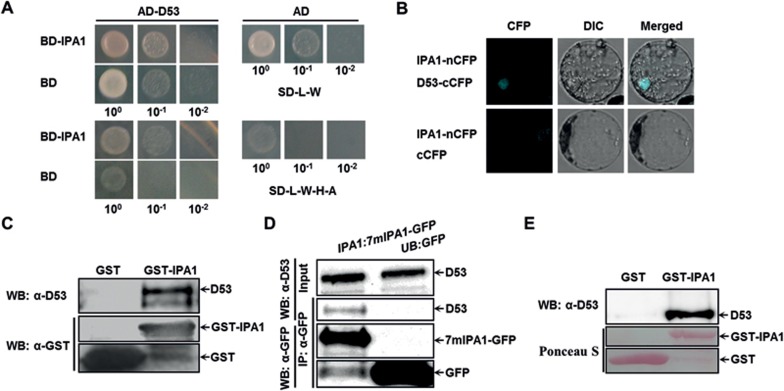
Interaction of IPA1 with D53 *in vitro* and *in vivo*. **(A)** Interaction between IPA1 and D53 revealed by yeast two-hybrid. IPA1 was fused with the GAL4 binding domain (BD) and D53 with the GAL4 activation domain (AD). The yeast clones were grown on the SD medium without leucine, tryptophan, histone and adenine (SD-L-W-H-A) with dilutions to 10^−1^ and 10^−2^. Yeast grown on the SD medium without leucine and tryptophan (SD-L-W) were used as a loading control. **(B)** Interaction between IPA1 and D53 revealed by the BiFC assay in rice protoplasts. IPA1 was fused with the N-terminal of CFP and D53 with the C-terminal of CFP. **(C)** Interaction between IPA1 and D53 revealed by GST pull-down with GST-IPA1 purified from bacteria and D53 extracted from rice calli. D53 was detected by rabbit polyclonal antibodies anti-D53 and GST by mouse monoclonal antibody anti-GST. **(D)** *In vivo* interaction between 7mIPA1-GFP and D53 revealed by the Co-IP assay in rice protoplasts. Proteins were extracted from *ProIPA1:7mIPA1-GFP* or *ProUB:GFP*. D53 was detected by rabbit polyclonal antibodies anti-D53 and GFP by mouse monoclonal antibody anti-GFP. **(E)** Interaction between IPA1 and D53 revealed by the GST pull-down assay with HisTrx-D53 and GST-IPA1 purified from bacteria. D53 was detected by rabbit polyclonal antibodies anti-D53 and GST by Ponceau S staining.

To further study the roles of IPA1 in the SL signaling pathway, we first tested whether mRNA or protein levels of *IPA1* are regulated by SLs and found that they showed no significant changes after GR24 treatment (Supplementary information, Figure S3). Similarly, we also found that the protein levels of IPA1 showed no differences between wild-type Nipponbare and SL-related mutants (Supplementary information, Figure S4). These results suggest that SLs do not affect the transcript and protein levels of *IPA1*. We then asked whether IPA1 could physically interact with D53, the repressor in the SL signaling pathway, in several ways. We first carried out a yeast two-hybrid assay in which we fused IPA1 to GAL4 DNA binding domain (BD) and D53 to GAL4 activation domain (AD) to form BD-IPA1 and AD-D53. We found that expression of BD-IPA1 and AD-D53 in co-transformed yeast cells could activate the expression of the *ADE* reporter gene to allow the transformed yeast cells to grow on the SD medium without adenine, indicating an interaction between IPA1 and D53 (Figure 2A). We confirmed an interaction between IPA1 and D53 by bimolecular fluorescence complementation (BiFC), GST-pull down using rice calli, and co-immunoprecipitation (Co-IP) assays (Figure 2B-2D). Finally, the direct interaction between the two proteins was further demonstrated in an *in vitro* pull down assay using proteins purified from a prokaryotic expression system (Figure 2E), indicating that no other plant protein is required for the interaction between IPA1 and D53. Taken together, these results allow us to conclude that IPA1 directly interacts with D53 in rice.

### D53 inhibits the transcriptional activator function of IPA1

**Figure 3:**
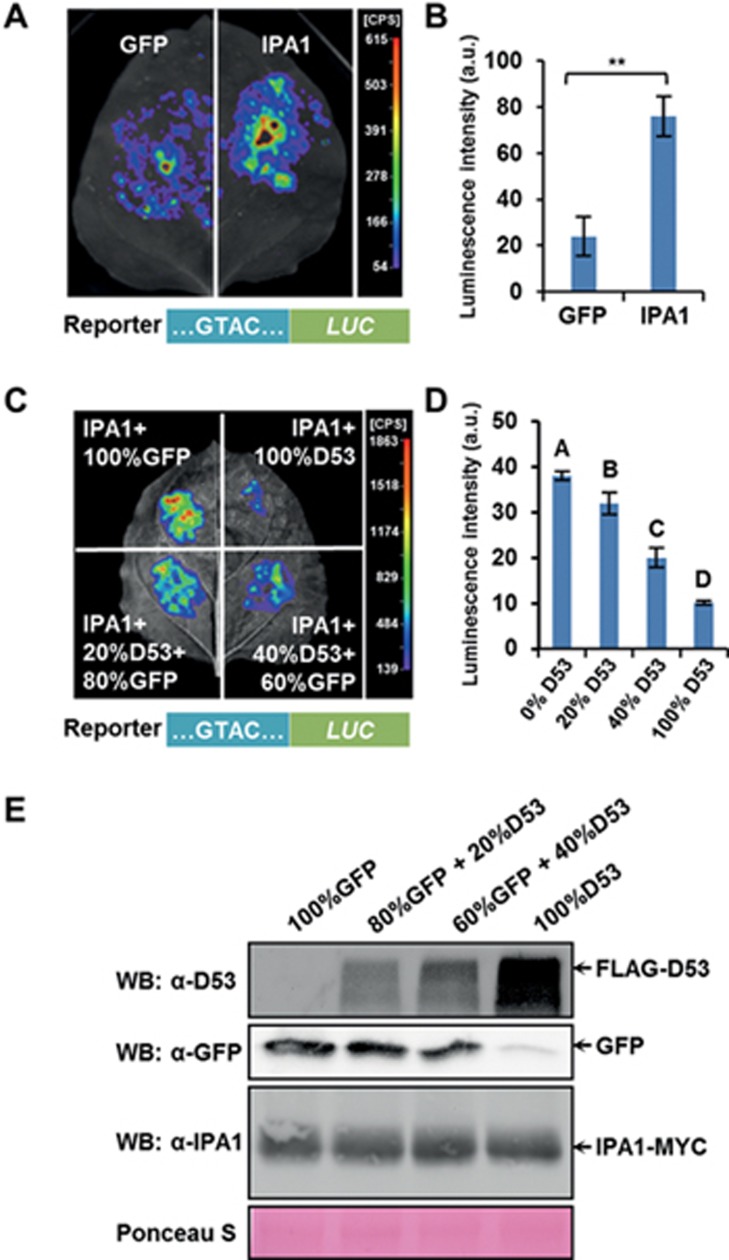
D53 represses the transcriptional activation activity of IPA1. **(A)** Transcriptional activity assay in tobacco leaves, showing that IPA1 promotes the expression of the reporter gene *Luciferase* (*LUC*) driven by the GTAC-containing promoter. *A. tumefaciens* transformed with *Pro35S:IPA1-MYC*, *Pro35S:GFP*, *Pro35S:GUS*, and *ProGTAC:LUC* were mixed and injected into tobacco leaves. D-luciferin was used as the substrate of LUC. **(B)** Statistical analysis of **(A)**. Values are means ± SD (*n* = 3). The asterisks represent significant difference determined by Student's *t* test. ^**^P < 0.01. **(C)** Transcriptional activity assay in tobacco, showing that D53 represses the transcriptional activation activity of IPA1. *A. tumefaciens* transformed with *Pro35S:IPA1-MYC*, *Pro35S:GFP*, *ProGTAC:LUC*, *Pro35S:GUS*, and *Pro35S:FLAG-D53* were mixed and injected into tobacco leaves. D-luciferin was used as the substrate of LUC. **(D)** Statistical analysis of **(C)**. Values are means ± SD (*n* = 3). Different letters at top of each column indicate a significant difference at *P* < 0.05 determined by Tukey's HSD test. **(E)** Protein levels in different infiltration combinations in **(C)**. D53 was detected by rabbit polyclonal antibodies anti-D53, GFP by mouse monoclonal antibody anti-GFP, and IPA1 was detected by rabbit polyclonal antibodies anti-IPA1. Ponceau S staining was used as loading control. See also Supplementary information, Figure S5.

D53 contains three EAR motifs in its C-terminal part and interacts with the transcriptional co-repressor TPL/TPR proteins^38,40^. However, the molecular mechanisms downstream of D53 remain elusive. The interaction between D53 and IPA1 raises the possibility that D53 could suppress the transcriptional activation activity of IPA1. We therefore investigated the effect of D53 upon the transcriptional activity of IPA1 using a reporting system in tobacco (*Nicotiana benthamiana*). In this system, the *LUC* (*Luciferase*) reporter gene was driven by a DNA sequence containing the SQUAMOSA PROMOTER BINDING PROTEIN (SBP) binding element GTAC. When co-infiltrating *Agrobacterium* hosts expressing IPA1-MYC together with the reporter construct, the expression of *LUC* was dramatically enhanced (Figure 3A and 3B; Supplementary information, Figure S5A). This indicates that IPA1 can activate the transcription of *LUC*. We then co-expressed FLAG-D53 and IPA1-MYC in tobacco leaves where we found these two proteins could interact with each other (Supplementary information, Figure S5B). Finally, we infiltrated *Agrobacterium* hosts expressing IPA1-MYC with varying ratios of GFP to FLAG-D53 into tobacco leaves and found that the activity of LUC gradually decreased in accordance with the amount of added FLAG-D53 proteins (Figure 3C and 3D; Supplementary information, Figure S5C). This was further confirmed by the western blotting analysis (Figure 3E), thus demonstrating that D53 could inhibit the transcriptional activation activities of IPA1.

### Requirement of D53-IPA1 interaction for D53 repression

**Figure 4:**
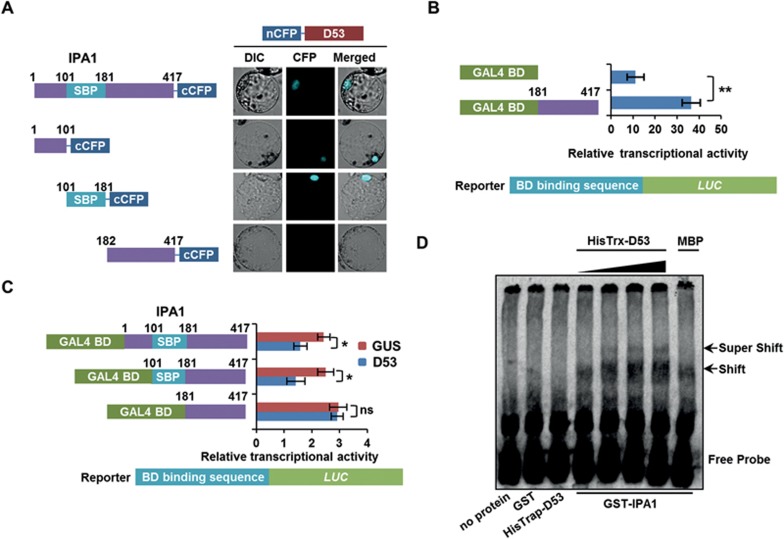
Interaction of IPA1 and D53 inhibits IPA1 transcriptional activation activity. **(A)** Interaction between the N-terminal, the SBP domain or the C-terminal of IPA1 and D53 revealed by the BiFC assay in rice protoplasts. The N-terminal, SBP domain or C-terminal of IPA1 were fused with cCFP and D53 was fused with nCFP. **(B)** The C-terminal of IPA1 was sufficient for the transcriptional activation activity of IPA1. Values are means ± sem (*n* = 3). The double asterisks represent significant difference determined by Student's *t* test at *P* < 0.01. **(C)** Transcriptional activity assay in rice protoplasts, showing that the N-terminal and the SBP domain are necessary for the D53-mediated repression of IPA1 transcriptional activation activity. Values are means ± SD (*n* = 3). The asterisk represents significant differences determined by Student's *t* test at *P* < 0.05. ns, no significant difference. GUS was used as a control. The transcriptional activation activity of full length IPA1 or IPA1-ΔN, which could bind to D53, was repressed by added D53 compared with the control. However, the activity of IPA1-ΔN-ΔSBP, which could not bind to D53, was not influenced by added D53 compared with control. **(D)** EMSA assay, showing that D53 does not affect the DNA binding activity of IPA1. The shift bands indicated the binding of GST-IPA1 to the probe containing GTAC element, and the super shift bands indicated the binding of GST-IPA1 together with HisTrx-D53, which was enhanced by adding HisTrx-D53, but not by MBP.

To investigate which domains of the two protein molecules are required for the direct interaction between IPA1 and D53, we first dissected their interaction domains through a domain mapping approach. Since the SBP domain of IPA1 is responsible for DNA binding activity and the C-terminal region functions as the activation domain^47^, we assayed the binding ability of the N-terminal, SBP-box and C-terminal regions of IPA1 with D53 by the BiFC assay. We found that both the N-terminal and the SBP-box of IPA1 could interact with D53 in rice protoplasts, but its C-terminal could not (Figure 4A). This is consistent with our previous finding that the activation domain of IPA1 is located at the C-terminal using the X-gal assay in yeast cells^47^. Here, we further found that the IPA1 C-terminal domain is sufficient to activate the reporter gene in the rice protoplast system (Figure 4B) and that D53 could repress the activation activity of the full length IPA1 and IPA1-ΔN but not that of IPA1-ΔN-ΔSBP (Figure 4C). Thus, the direct interaction between D53 and IPA1 was required for the suppression of IPA1.

Considering that D53 might inhibit the function of IPA1 through affecting the DNA binding activity or the transcriptional activation activity of IPA1, we wanted to investigate whether D53 could repress the DNA binding activity of IPA1 in an electrophoretic mobility shift assay (EMSA). In the presence of the HisTrx-D53 fusion protein, the DNA binding activity of IPA1 was not affected and a super-shifted band could be observed (Figure 4D), indicating that the interaction between D53 and IPA1 did not affect the DNA binding activity of IPA1. Taken together, these results demonstrate that D53 forms a complex with IPA1 and inhibits the transcriptional activation activity of IPA1 without affecting the DNA binding activity of IPA1.

### IPA1 binds to the promoter of *D53* and regulates its transcription

**Figure 5:**
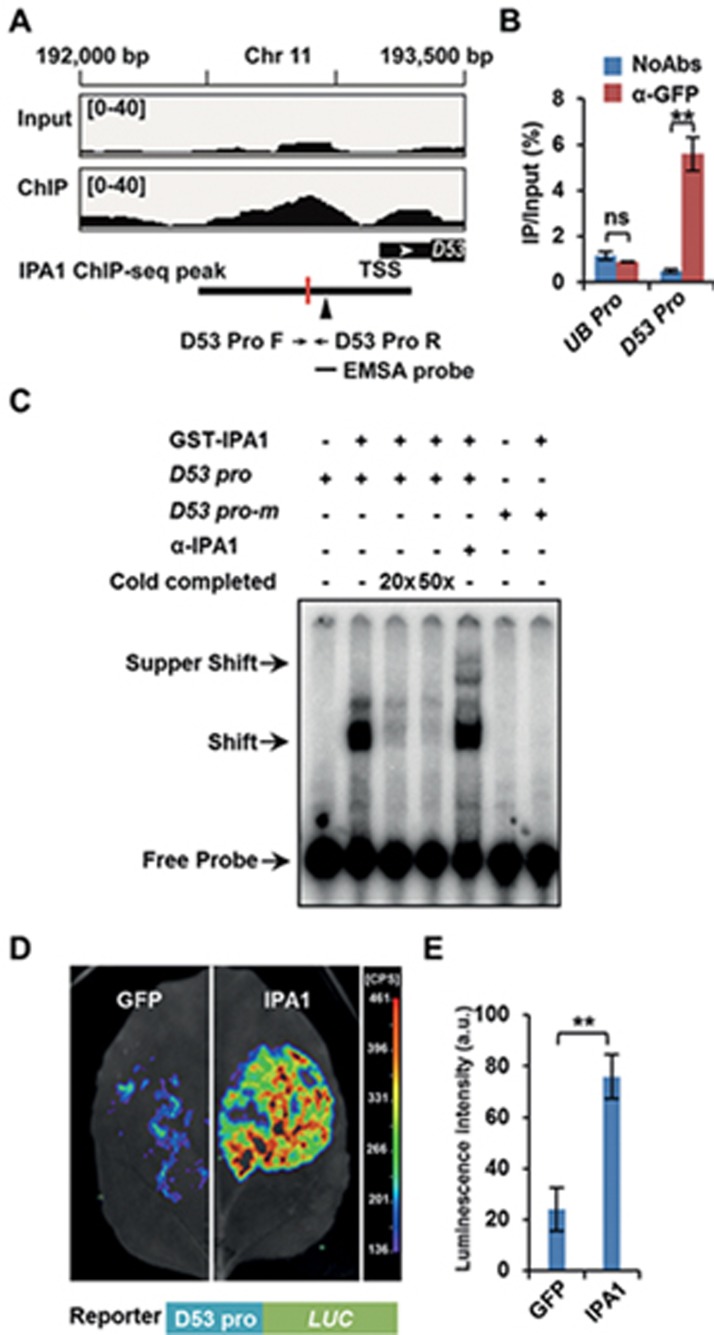
IPA1 binds to the *D53* promoter and regulates D53 expression. **(A)** IPA1 binding profile in the promoter of *D53*. The solid arrowhead refers to the GTAC around the peak summit, and red vertical line to peak summit. Primer pairs *D53-ProF* and *D53-ProR* (Supplementary information, Table S1) were used for ChIP-qPCR. The probe was used in EMSA. TSS, transcription start site. **(B)** Validation of IPA1 direct binding sites in the *D53* promoter by ChIP-qPCR analysis. Values are means ± sem (*n* = 3). The double asterisk represents significant difference determined by the Student's *t* test at *P* < 0.01; ns, no significant difference. **(C)** Direct binding of IPA1 to the *D53* promoter in the EMSA assay. The 20- and 50-fold excess non-labeled probes were used for competition. The *D53 pro-m* is a mutated version of *D53 pro* probe with the SBP binding motif GTAC changing to ATAC. **(D)** Transcriptional activity assay in tobacco leaf, showing that IPA1 could enhance the expression of the *D53* promoter-drived *LUC* reporter. *A. tumefaciens* transformed with *Pro35S:IPA1-MYC*, *Pro35S:GFP*, and *ProD53:LUC* were mixed and injected into tobacco leaves. D-luciferin was used as the substrate of Luciferase. **(E)** Statistical analysis of **(D)**. Values are means ± SD (*n* = 3). The asterisk represents significant difference determined by Student's *t* test. ^**^P < 0.01.

Although SLs play key roles in the regulation of shoot branching and other biological functions, only a few early SL-responsive genes have been identified^39,50^. In rice, the *D53* transcript is rapidly induced upon GR24 treatment^38,39^. By analyzing IPA1 Chromatin Immunoprecipitation (ChIP)-seq data^47^, we found one IPA1 binding peak on the promoter region of *D53* in shoot apexes with the peak summit 286 bp upstream of the *D53* transcription start site (TSS). This peak contains a GTAC element which may be responsible for IPA1 binding (Figure 5A). Thus, we conducted ChIP-qPCR assays using specific primers and an anti-GFP antibody in the shoot base of *ProIPA1:7mIPA1-GFP* transgenic plants, and found that the IPA1 enrichment levels at the *D53* promoter were significantly higher than at the *Ubiquitin* promoter (Figure 5B). To test whether IPA1 could directly bind to the GTAC motif in the *D53* promoter, we performed an EMSA and found that GST-IPA1 could dramatically reduce the migration of the 59-bp probe from the *D53* promoter and the added IPA1 antibodies could intensify the retardation, indicating that IPA1 could directly bind to the *D53* promoter (Figure 5C).

Our findings that the *D53* promoter is the target of IPA1 suggest that IPA1 might promote the expression of *D53*. We tested this in a transient expression assay in *N. benthamiana* using the *D53* promoter fused to the *LUC* reporter gene. We co-infiltrated *N. benthamiana* leaves with the *35S:IPA1-MYC* construct and the *ProD53:LUC* reporter and found that the luminescence intensity was dramatically elevated (Figure 5D and 5E), indicating that IPA1 could directly bind to the *D53* promoter and activate its expression.

### Induction of *D53* transcript by SL depends on IPA1 and is part of a feedback loop

**Figure 6:**
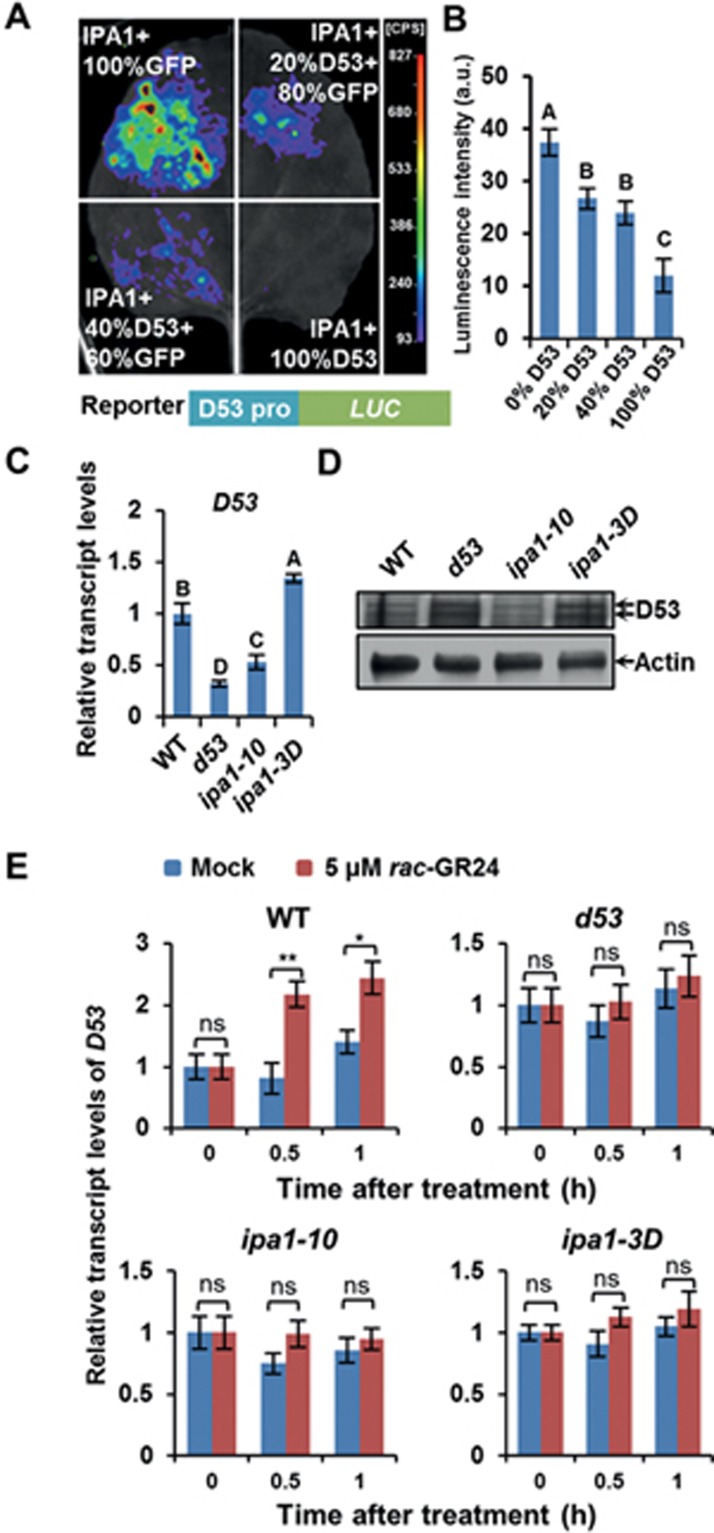
D53 and IPA1 form a feedback regulation loop in SL signaling. **(A)** Transcriptional activity assay in tobacco, showing that D53 represses the IPA1-drived activation of the *D53* promoter. *A. tumefaciens* transformed with *Pro35S:IPA1-MYC*, *Pro35S:GFP*, *ProD53:LUC*, and *Pro35S:FLAG-D53* were mixed and injected into tobacco leaves. D-luciferin was used as the substrate of LUC. **(B)** Statistical analysis of **(A)**. Values are means ± SD (*n* = 3). Different letters at top of each column indicate a significant difference at *P* < 0.05 determined by Tukey's HSD test. **(C)** *D53* transcript levels in WT, *d53* and *ipa1* mutants. Values are means ± sem (*n* = 3). Different letters at top of each column indicate a significant difference at *P* < 0.05 determined by Tukey's HSD test. **(D)** D53 protein levels in WT, *d53* and *ipa1* mutants. D53 was detected by rabbit polyclonal antibodies anti-D53. Actin was used as the loading control. **(E)** Mutations in IPA1 disrupt SL-induced *D53* transcription after *rac*-GR24 treatment. Values are means ± sem (*n* = 3). Statistical differences between mock and treatment at same time points were determined by Student's *t* test. ^*^P < 0.05, ^**^P < 0.01; ns, no significant difference.

Based on the findings that D53 could inhibit the transcriptional activation activity of IPA1 and that IPA1 could directly regulate *D53* expression, we explored the possibility of whether the D53 protein could inhibit its own transcription through IPA1. Thus using the transient expression system expressing the *ProD53:LUC* reporter construct in *N. benthamiana*, we co-infiltrated *Agrobacterium* hosts expressing IPA1-MYC together with varying ratios of *Agrobacterium* hosts expressing GFP and FLAG-D53. This revealed that the activity of LUC gradually decreased when more FLAG-D53 was added (Figure 6A and 6B). In accord with the result of the transient assay, disruption of IPA1 in *ipa1-10* leads to downregulated transcript and protein levels of *D53* compared with that in the wild-type plant, whereas *ipa1-3D*, the gain-of-function mutant, displays elevated levels (Figure 6C and 6D). These results demonstrate that IPA1 can promote the expression of *D53*, which in turn inhibits the transcriptional activation activity of IPA1, thus forming a negative feedback loop.

We further investigated whether IPA1 is responsible for the SL-induction of *D53* transcripts by treating wild-type, *d53*, *ipa1-10*, and *ipa1-3D* seedlings with *rac*-GR24. As shown in Figure 6E, upon the *rac*-GR24 treatment the *D53* transcripts were significantly increased in WT plants but they showed no significant difference to the mock treated *d53*, *ipa1-10*, and *ipa1-3D* plants, indicating that the induction of the *D53* transcription by SL depends on normal expression of *IPA1*. This result is consistent with the fact that the shoot branching phenotypes of *ipa1-10* and *ipa1-3D* plants are both insensitive to an exogenous *rac*-GR24 treatment. Taken together, these results show that in the rice SL signaling pathway D53 represses the transcriptional activation activity of IPA1 to down regulate its downstream genes, and this in turn affects the *D53* transcription, forming a feedback-loop in response to SLs.

### Cross talk between miRNA156 and the SL signaling pathway

**Figure 7:**
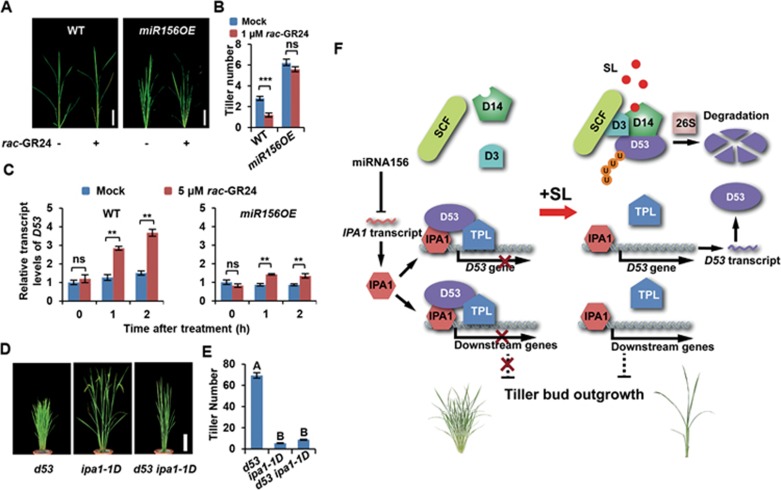
Overexpression of miRNA156 compromises SL response. **(A)** Gross morphologies of wild-type and miRNA156 overexpression (*miR156OE*) plants with or without *rac*-GR24 treatment. Seedlings were treated with 1 μM *rac*-GR24 (+) or mock (–). Bar = 5 cm. **(B)** Statistical analysis of tiller number in **(A)**. Values are means ± sem (*n* = 5). The asterisks represent significant difference determined by Student's *t* test. ^***^P < 0.001; ns, no significant difference. **(C)** Overexpression of miRNA156 disrupts SL-induced *D53* transcription after *rac*-GR24 treatment. Values are means ± sem (*n* = 3). Statistical differences between mock and treatment at the same time points were determined by Student's *t* test. ^**^P < 0.01; ns, no significant difference. **(D)** Gross morphologies of *d53*, *ipa1-1D*, and *d53 ipa1-1D* double mutant plants. Bar = 20 cm. **(E)** Statistical analysis of **(D)**. Values are means ± sem (*n* = 8). Different letters at top of each column indicate a significant difference at *P* < 0.05 determined by Tukey's HSD test. **(F)** A proposed model of the IPA1-mediated SL signaling pathway. In the absence of SLs, the D53 protein binds to IPA1, and together with TPL/TPR proteins represses the transcriptional activity of IPA1. In the presence of SLs, perception of SL leads to degradation of D53 by the proteasome system, which in turn releases the repression of IPA1-regulated gene expression and leads to SL response.

IPA1 is post-transcriptionally controlled by miRNA156 and miRNA529^45,46^. In miRNA156 overexpressing (*miR156OE*) plants, the tillering number is dramatically increased (Supplementary information, Figure S6) and *SPL* genes including *IPA1* are remarkably down-regulated^56,57^. When treated with *rac*-GR24, the inhibition of bud outgrowth in *miR156OE* plants was considerably weaker than in the wild type (Figure 7A, 7B). Moreover, the induction of the *D53* transcription by *rac*-GR24 was strongly impaired in *miR156OE* plants (Figure 7C). These data suggest that *IPA1* may be the common target of miRNA156 and the SL signaling pathway, and that the impairment of the SL signaling in *miR156OE* plants is probably due to the diminished expression of *IPA1*.

To further investigate the role of IPA1 in SL signaling, we constructed the *ipa1-1D d53* double mutant by crossing *Ri22*, an *ipa1-1D* allele, with *d53*. Genetic analysis showed that *ipa1-1D* could significantly suppress the high tillering phenotype of *d53* (Figure 7D and 7E). Similarly, we generated the *ipa1-1D d27* and *ipa1-1D d10* double mutants, and found that *ipa1-1D* could repress the high tillering phenotype of both *d27* and *d10* (Supplementary information, Figure S7), consistent with the previous observation that the overexpression of *IPA1* could suppress the high-tillering phenotype of *d10*^58^. These genetic data substantiate the finding that IPA1 works downstream of D53 in the SL signaling pathway, where D53 functions as a transcriptional repressor; IPA1 functions in downstream of D53 as a transcription factor to regulate rice tillering as well as enhance *D53* expression as a feedback mechanism (Figure 7F).

## Discussion

D53 has been identified as a key transcriptional repressor in SL signaling based upon the finding that D53 can be ubiquitinated and degraded by the SCF^D3^ complex in an SL-dependent manner and that D53 contains three EAR motifs and physically interact with transcriptional co-repressor TPL/TRP proteins^38,39,40,59,60^. However, the transcription factors targeted by D53 remain elusive and the molecular mechanism of the transcriptional regulation by D53 are still in debate^44,61^. One major concern is that although SLs regulate multiple developmental processes, few genes have been identified to respond to SL treatment^50^. Significantly, the transcription of *D53* is induced within 1 h of SL treatment and *D53* expression levels are greatly repressed in a series of *d* mutants^38^. In this study, we demonstrate that D53 can physically interact with IPA1, a member of the SPL family transcription factors, and inhibit its transcriptional activation activity. In the *ipa1* loss-of-function and overexpression plants, GR24 treatment could neither inhibit bud outgrowth nor induce the expression of *D53* transcripts, indicating that IPA1 functions downstream of D53 in the SL signaling pathway. Therefore, we propose a working model, in which IPA1 functions as the transcription factor that works directly downstream of D53 in the SL signaling pathway (Figure 7F). In the absence of SLs, D53 protein binds to IPA1, and together with TPL/TPR proteins represses the transcriptional activity of IPA1. In response to SLs, D53 is degraded by the proteasome system, which in turn releases the repression of IPA1-regulated gene expression resulting in the SL response. Moreover, we found that IPA1 can directly bind to the *D53* promoter and upregulate *D53* expression, forming a negative feedback regulation loop.

Feedback loops are considered to play important roles in different signaling pathways^62,63,64^. In the SL biosynthetic and signaling pathways, several key components are tightly regulated through negative feedback loops^10,38,39,41,50,65^. In *Arabidopsis thaliana*, the key biosynthetic genes *MAX3* and *MAX4* are repressed by GR24 treatment but show elevated expression in *max* mutants^41,50^. AtD14 has been shown to undergo 26S-proteasome-dependent degradation after GR24 treatment, which would effectively limit the duration and intensity of SL signaling^65^. In rice, the transcript levels of *D53* in *d27*, *d17*, *d10*, *d3*, and *d14* are lower than that in wild type, in contrast, the D53 protein accumulates in all *d* mutants whereas the *D53* transcript level increases but D53 protein is degraded rapidly after the SL treatment^38^. This opposite phenomenon raises the possibility that a negative feedback loop may exist to regulate the activity of D53 *in planta*. However, the specific mechanism of these feedback regulations in SL biosynthesis and signaling is still elusive. Here we found that IPA1 can directly bind to the promoter of *D53* and activate *D53* expression and that the D53 protein can form a complex with IPA1 and repress transcriptional activation of *D53*. Thus, the D53 protein may directly inhibit *D53* mRNA transcription through interacting with IPA1 and repressing its activity. This feedback regulation would cause a substantial drop in SL signal transduction and would be important for SLs to precisely regulate plant development to enable a rapid response to environmental stimulants.

The mechanism of SL perception and signaling mediated by the SL receptor D14, the F-box protein D3 and the repressor D53 are conserved across monocotyledonous and dicotyledonous species^6,9,13,34,37,38,41,43^. In *Arabidopsis thaliana* and pea, *BRC1* is considered as one important downstream gene in the SL signaling pathway, which is strongly induced after GR24 treatment to negatively regulate the development of rosette and cauline branches^49,51,52^. Meanwhile, *BRC1* transcripts are down-regulated in *max2* and *max3* mutants, but up-regulated in *smxl6/7/8* triple mutants^41^. In rice, it has been reported that mutants of *OsTB1*, the rice ortholog of *BRC1*, are insensitive to GR24 for tiller outgrowth^66^, leading to the assumption that the downstream SL signaling pathway may also be conserved. However, in the 2-week-old rice seedling, *OsTB1* expression shows no induction following 2 μM *rac*-GR24 treatment in shoot bases containing the shoot apical meristem (SAM), axillary buds, young leaves and nodes^66^. Further understanding is needed of transcription regulation of *OsTB1* in a more specific tissue, especially in the bud whose elongation is regulated by SL. In our previous work, we showed that IPA1 could bind to the promoter of *OsTB1* and regulate its expression^46^. But in *Arabidopsis thaliana*, loss-of-function mutants of different *SPL* genes are sensitive to SL treatment, and no evidence has been found of *BRC1*'s regulation by SPL proteins, suggesting that AtSPLs are probably not involved in *BRC1* induction by SLs^67^. It seems that the downstream SL signaling pathway is not fully conserved between monocotyledonous and dicotyledonous plants, and that Os*SPL* genes and *OsTB1* may have diverse functions with *AtSPL* genes and *BRC1* in response to SLs. However, further evidence is required to support this hypothesis. Moreover, in *Arabidopsis thaliana*, it is reported that SLs modulate polar auxin transport through the endocytosis of PIN-FORMED1 (PIN1)^68,69^. PIN1 is localized to the plasma membrane and it is rapidly depleted after SL treatment, independently of protein synthesis^69^. In rice, it is reported that basipetal polar auxin transport is elevated in *d27*^14^, and that IAA level is increased in *d3*^28^, suggesting that the crosstalk between SL and auxin, two key plant hormones for plant development, is of great importance in both species. However, the mechanism of this crosstalk is still obscure. From IPA1 ChIP-seq data, we found that IPA1 binding sites are located on the promoter of *PIN1b*^47^, but whether and how IPA1 and PIN1b are involved in the crosstalk between auxin and SL needs further study.

The high tillering phenotypes of *d* mutants have been well studied, but there are other significant phenotypes such as dwarfism and low fertility that need further investigation^12,13,14^. As a pleiotropic regulator, IPA1 regulates many important phenotypes, such as tiller number, plant height and panicle morphology in rice^45,46^. More importantly, IPA1 has been widely used in breeding elite rice varieties due to its great potential in improving rice yield^45,54,55^. It will be impoartant to have an in-depth characterization of the regulatory network of IPA1 and D53 to reach a better understanding of rice architecture and provide a powerful tool and new targets for molecular breeding in rice.

## Materials and Methods

### Plant materials

Rice (*Oryza sativa* L.) ssp. *japonica* Nipponbare, *ProIPA1:7mIPA1-GFP* transgenic line, ZH11, *ipa1-10*, *ipa1-11*, *ipa1-3D*, *ipa1-4D*, *miR156OE*, *Ri22*, *d53* and other mutants were grown either in the green house under 16 h light and 8 h dark at 28 °C or the experimental field of the Institute of Genetics and Developmental Biology. Double mutants of *ipa1-1D d53*, *ipa1-1D d10*, and *ipa1-1D d27* were generated from crosses between *Ri22* (*japonica*), an *ipa1-1D* cultivar, and *d53*, *d10*, and *d27* mutants, respectively.

### TILLING

TILLING was performed in a population of 6 000 EMS-mutagenized M2 rice plants in the ZH11 background (*Oryza sativa* L. *japonica*) using PCR primer pairs targeted to a conserved domain of *IPA1*. Crude CEL1 endonuclease was extracted from celery^70,71^, and used for the cleavage of PCR products at mismatched positions of re-annealed heteroduplexes. Point mutations were detected by capillary electrophoresis using AdvanCEFS96 (Advanced Analytical Technologies, USA).

### Protein interaction analysis

The primer pairs used in protein interaction analysis are listed in Supplementary information, Table S1. For the yeast two-hybrid assay, the *IPA1* coding sequence (CDS) and *D53* CDS were inserted into pGBKT7 and pGADT7 respectively. The Matchmaker^®^ Gold Yeast Two-Hybrid System (Clontech) was used to test the interaction between IPA1 and D53 following the manufacturer's instructions. For the BiFC assay, the *D53* CDS and different truncations of the *IPA1* CDS were amplified and cloned into pUC-SCYNE(R) and pUC-SCYCE respectively^72^. The BiFC assay was performed as described previously^47^. For the Co-IP assay, total proteins were extracted from *ProIPA1:7mIPA1-GFP* or *ProUB:GFP* transgenic calli according to the method described previously^38^. Following the manufacturer's instructions, 30 μl of the agarose-conjugated anti-GFP monoclonal antibody (MBL, D153-8) were added into 500 μl total extracted proteins and incubated at 4 °C for 3 h with gentle rotation. The beads were washed three times with 350 μl extraction buffer, and eluted with 30 μl SDS-PAGE sample buffer. Immunoblotting was performed as described^38,47^. For the GST pull-down, purified GST, GST-IPA1, HisTrx-D53 and proteins extracted from rice calli were used in the assay, which was performed as described^38^. HisTrx-D53 fusion protein and D53 protein from rice calli were detected by rabbit polyclonal antibodies anti-D53^38^ and GST fusion proteins by Ponceau S or a monoclonal mouse antibody anti-GST (Abmart, M20007).

### RNA isolation and quantitative real-time PCR

RNA isolation, reverse transcription and real-time PCR were performed as described previously^47^. Primer pairs used in real-time PCR were listed in Supplementary information, Table S1.

### Expression and purification of fusion proteins

The full-length CDS of *IPA1* or *D53* was amplified by the primer pairs listed in Supplementary information, Table S1 and cloned into the *Escherichia coli* expression vector pGEX-6p-1 (GE Healthcare) or pDEST51. Expression and purification of fusion proteins were conducted as described^38,47^.

### ChIP-qPCR assay

The ChIP-qPCR using the *ProIPA1:7mIPA1-GFP* transgenic seedlings was performed according to the method described previously^47,73^. PCR reactions were performed in triplicate for each sample, and expression levels were normalized to the input sample for enrichment detection. The fold enrichment was calculated against the *Ubiquitin* promoter. No addition of antibodies (NoAbs) was served as a negative control.

### EMSA

The probe sequences are listed in Supplementary information, Table S1. Probe labelling and EMSA were performed as described previously^47^.

### Transcriptional activity assay in tobacco leaf

To generate the *ProGTAC:LUC* and *ProD53:LUC*, synthesized GTAC sequence and *D53* promoter were cloned into the pCAMBIA1301-LUC vector. To generate the *Pro35S:IPA1-MYC* and *Pro35S:FLAG-D53*, CDS of *IPA1* and *D53* were cloned to the 1300-MYC and pDEST1300-FLAG vectors, respectively. The plasmids used in this study were transformed to *Agrobacterium tumefaciens* strain EHA105. *p19* was used to suppress RNA silencing, pCAMBIA1301 for GUS expression as an internal control. Agroinfiltration and luciferase imaging were performed as described previously^74^. GUS activities were measured with 4-methylumbelliferyl-β-D-glucuronide (Sigma) according to the manufacturer's instructions.

### Transcriptional activity assay in rice protoplasts

The plasmids containing GAL4BD-IPA1, 35sLUC and pRTL or GAL4BD-IPA1-ΔN, 35sLUC and pRTL or GAL4BD-IPA1-ΔN-ΔSBP, 35sLUC and pRTL were introduced into rice leaf protoplasts as described^75^, with plasmids containing GAL4BD, pRTL and 35sLUC as a negative control. The assay was performed as described^47^.

### GR24 treatment

Two-week-old hydroponically cultured rice seedlings were grown in the climatic cabinet at 80% humidity, under 16 h light at 25 °C and 8 h dark at 16 °C. The seedlings were treated with 5 μM *rac*-GR24 (StrigoLab) or equal volume of acetone, then 0.5 cm shoot base samples were harvested at indicated time points. RNA isolation, cDNA synthesis, qRT-PCR, protein extraction, and immunoblotting were performed as described above.

### Accession numbers

Gene sequence used in this study can be found in the Rice Genome Annotation Project under accession numbers: LOC_Os08g39890 (*IPA1*) and LOC_Os11g01330 (*D53*).

## Author Contributions

XS, ZL, HY and JL conceived this project and designed all experiments. XS, ZL, HY, GS, JX, XM, YJ, GL, JD, XFY, CML and HL performed experiments. XS, ZL, HY, GX, YW and JL analyzed data. XS, ZL, HY and JL wrote the paper.

## Competing Financial Interests

The authors declare no competing financial interests.

## Supplementary Information

Supplementary information, Figure S1 TILLING lines of *IPA1* have no obvious phenotype.

Supplementary information, Figure S2 Genomic structure and mutant alleles of *IPA1*.

Supplementary information, Figure S3 Expression and protein levels of *IPA1* after *rac*-GR24 treatment.

Supplementary information, Figure S4 Protein levels of IPA1 in various *dwarf* mutants.

Supplementary information, Figure S5 Expression of the fusion proteins and interaction between IPA1 and D53 in tobacco leaves.

Supplementary information, Figure S6 High tillering phenotype of miRNA156-overexpressing transgenic plants.

Supplementary information, Figure S7 *ipa1-1D* represses the high tillering phenotype of *d10* and *d27*.

Supplementary information, Table S1 Primers used in this study
