# Supplementary material for: IPA1 functions as a downstream transcription factor repressed by D53 in strigolactone signaling in rice
Source: Cell Res. 2017 Aug 15;27(9):1128–41. doi: 10.1038/cr.2017.102 (PMC5587847; doi:10.1038/cr.2017.102)
Supplement: Supplementary information, Figure S1 — TILLING lines of IPA1 have no obvious phenotype. [file cr2017102x1.pdf]

A

Lines	Amino acid position	Amino acid substitution
S226	24	Gly to Asp
S228	5' UTR	
S230	57	Gly to Asp
S237	52	Gly to Val
S238	76	Gly to Ser

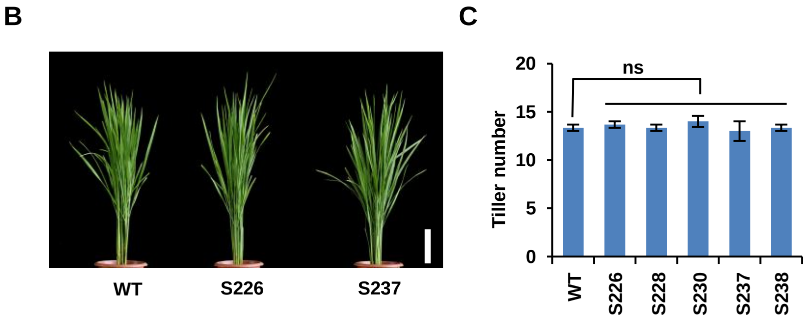

Figure S1 TILLING lines of *IPA1* have no obvious phenotype. **(A)** Mutation information for different IPA1 TILLING lines. **(B)** Gross morphologies of *IPA1* TILLING lines. Bar = 20 cm. **(C)** Statistical data of tiller numbers of different *IPA1* TILLING lines. Values are means ± SE (n = 3). Statistical difference was determined by Student's t test. ns, no significant difference.
