# Supplementary material for: IPA1 functions as a downstream transcription factor repressed by D53 in strigolactone signaling in rice
Source: Cell Res. 2017 Aug 15;27(9):1128–41. doi: 10.1038/cr.2017.102 (PMC5587847; doi:10.1038/cr.2017.102)
Supplement: Supplementary information, Figure S2 — Genomic structure and mutant alleles of IPA1. [file cr2017102x2.pdf]

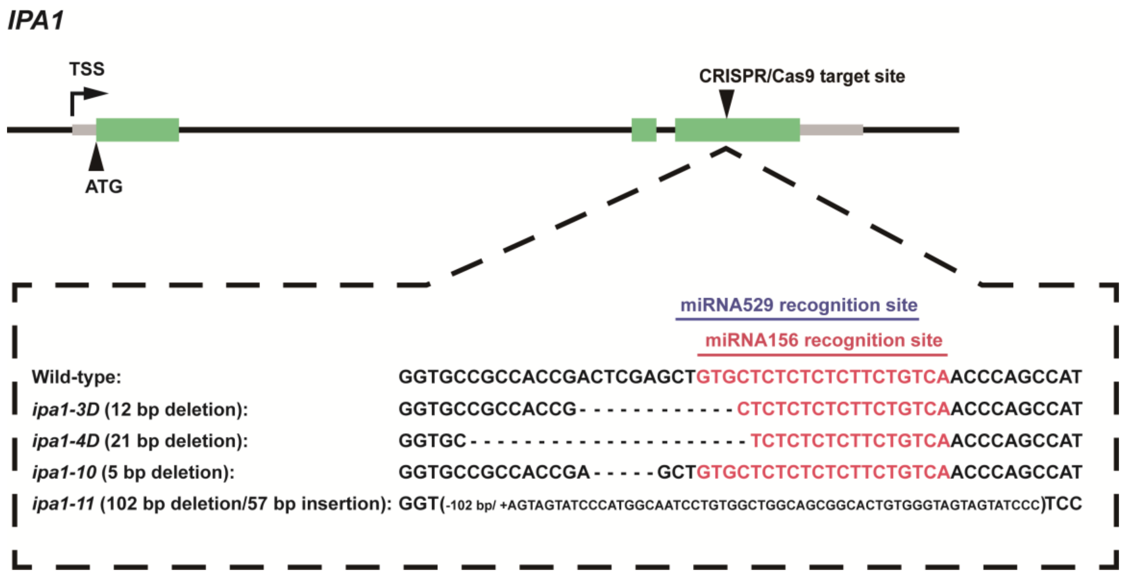

Figure S2 Genomic structure and mutant alleles of *IPA1*. Green boxes refer to the coding regions of the *IPA1* gene and gray boxes to untranslated regions. TSS, transcription start site. The gain-of-function mutants of *ipa1-3D* and *ipa1-4D* were resulted from the non-frame-shift deletion that destroys the miRNA recognition site, leading to the release from the miRNA regulation and an increased transcript level. The loss-of-function mutant of *ipa1-10* was resulted from a frame-shift deletion. The loss-of-function mutant of *ipa1-11* was resulted from a large fragment deletion.
