# Supplementary material for: IPA1 functions as a downstream transcription factor repressed by D53 in strigolactone signaling in rice
Source: Cell Res. 2017 Aug 15;27(9):1128–41. doi: 10.1038/cr.2017.102 (PMC5587847; doi:10.1038/cr.2017.102)
Supplement: Supplementary information, Figure S3 — Expression and protein levels of IPA1 after rac-GR24 treatment. [file cr2017102x3.pdf]

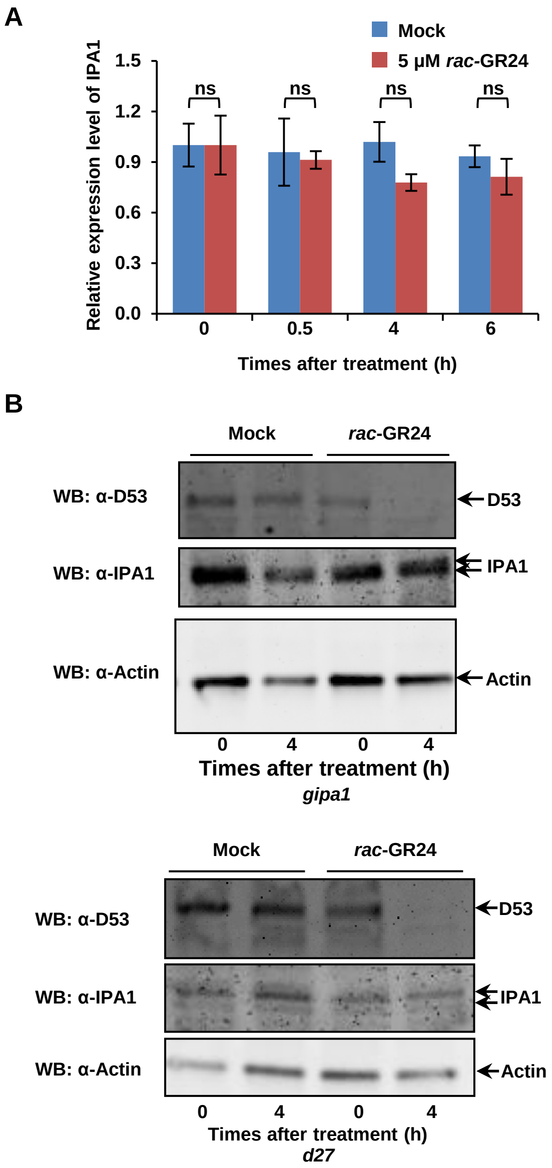

Figure S3 Expression and protein levels of *IPA1* after *rac*-GR24 treatment. **(A)** Expression levels of *IPA1* after *rac*-GR24 treatment. Rice *Actin1* was used as a reference. Values are means ± SE (n = 3). Statistical difference was determined by Student's t test (n = 3). ns, no significant difference. **(B)** Protein levels of IPA1 and D53 after *rac*-GR24 treatment in *ipa1-1D* genomic sequence transgenic line (*gipa1*) and *d27* mutant. Actin was used as the loading control.
