# Supplementary figures and images for: IPA1 functions as a downstream transcription factor repressed by D53 in strigolactone signaling in rice
Source: Cell Res. 2017 Aug 15;27(9):1128–41. doi: 10.1038/cr.2017.102 (PMC5587847; doi:10.1038/cr.2017.102)

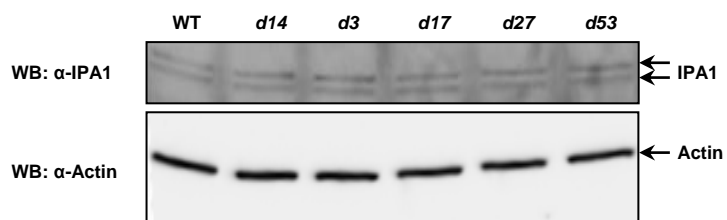

**Figure S4** Protein levels of IPA1 in various *dwarf* mutants. Actin was used as the loading control.

Supplement: Supplementary information, Figure S4 — Protein levels of IPA1 in various dwarf mutants. [file cr2017102x4.pdf]
