# Supplementary material for: IPA1 functions as a downstream transcription factor repressed by D53 in strigolactone signaling in rice
Source: Cell Res. 2017 Aug 15;27(9):1128–41. doi: 10.1038/cr.2017.102 (PMC5587847; doi:10.1038/cr.2017.102)
Supplement: Supplementary information, Figure S5 — Expression of the fusion proteins and interaction between IPA1 and D53 in tobacco leaves. [file cr2017102x5.pdf]

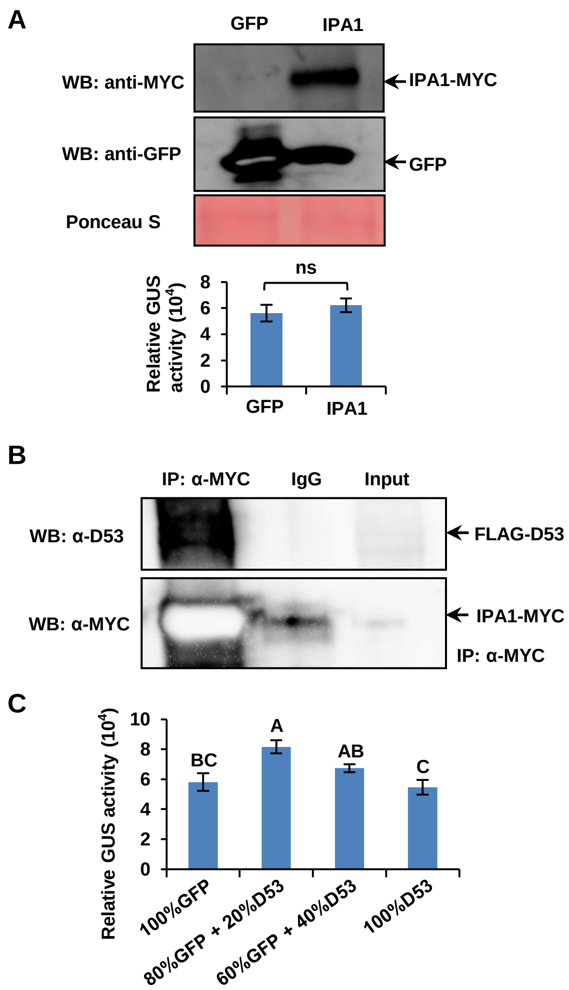

Figure S5 Expression of the fusion proteins and interaction between IPA1 and D53 in tobacco leaves. **(A)** Protein levels of the fusion proteins in the infiltration assay in Figure 3A. D53 was detected by rabbit polyclonal antibodies anti-D53, GFP by mouse monoclonal antibody anti-GFP, and IPA1 by rabbit polyclonal antibodies anti-IPA1. Ponceau S staining was used as the loading control. GUS activity was measured with the substrate 4-MUG and used as an internal control to indicate similar protein expression in different infiltration combinations. Values in the lower panel are means ± SD (n = 6). Statistical difference was determined by Student's t test. ns, no significant difference. **(B)** Interaction between IPA1 and D53 revealed by the Co-IP assay in tobacco leaves. Immunoprecipitation was performed with anti-MYC-agarose and immunoblotting was carried out with anti-D53 antibody and anti-MYC antibody, respectively. The IgG-agarose was used as a negative control. **(C)** The GUS activity in different infiltration combinations in Figure 3C. GUS activity was measured with the substrate 4-MUG and used as an internal control to indicate similar protein expression levels in different infiltration combinations. Values are means ± SD (n = 6). Different letters at top of each column indicate a significant difference at $P < 0.05$ determined by Tukey's HSD test.
