# Supplementary material for: IPA1 functions as a downstream transcription factor repressed by D53 in strigolactone signaling in rice
Source: Cell Res. 2017 Aug 15;27(9):1128–41. doi: 10.1038/cr.2017.102 (PMC5587847; doi:10.1038/cr.2017.102)
Supplement: Supplementary information, Figure S6 — High tillering phenotype of miRNA156-overexpressing transgenic plants. [file cr2017102x6.pdf]

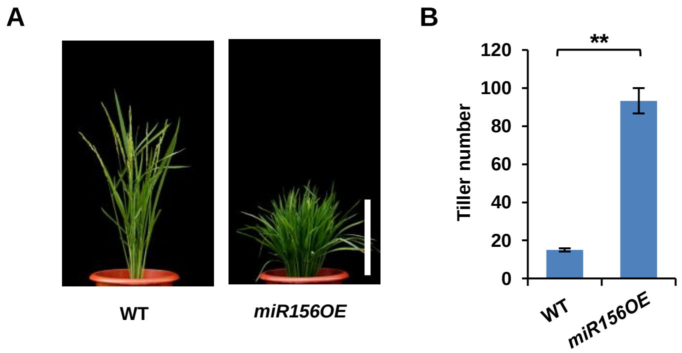

Figure S6 High tillering phenotype of miRNA156-overexpressing transgenic plants. **(A)** Gross morphology of miRNA156-overexpressing (*miR156OE*) transgenic plant. Bar = 20 cm. **(B)** Statistical analysis of tiller number of **(A).**Values are means ± SD ($n = 4$). The asterisks represent significant difference determined by Student's t test. **, $P < 0.01$.
