# Supplementary material for: IPA1 functions as a downstream transcription factor repressed by D53 in strigolactone signaling in rice
Source: Cell Res. 2017 Aug 15;27(9):1128–41. doi: 10.1038/cr.2017.102 (PMC5587847; doi:10.1038/cr.2017.102)
Supplement: Supplementary information, Figure S7 — ipa1-1D represses the high tillering phenotype of d10 and d27. [file cr2017102x7.pdf]

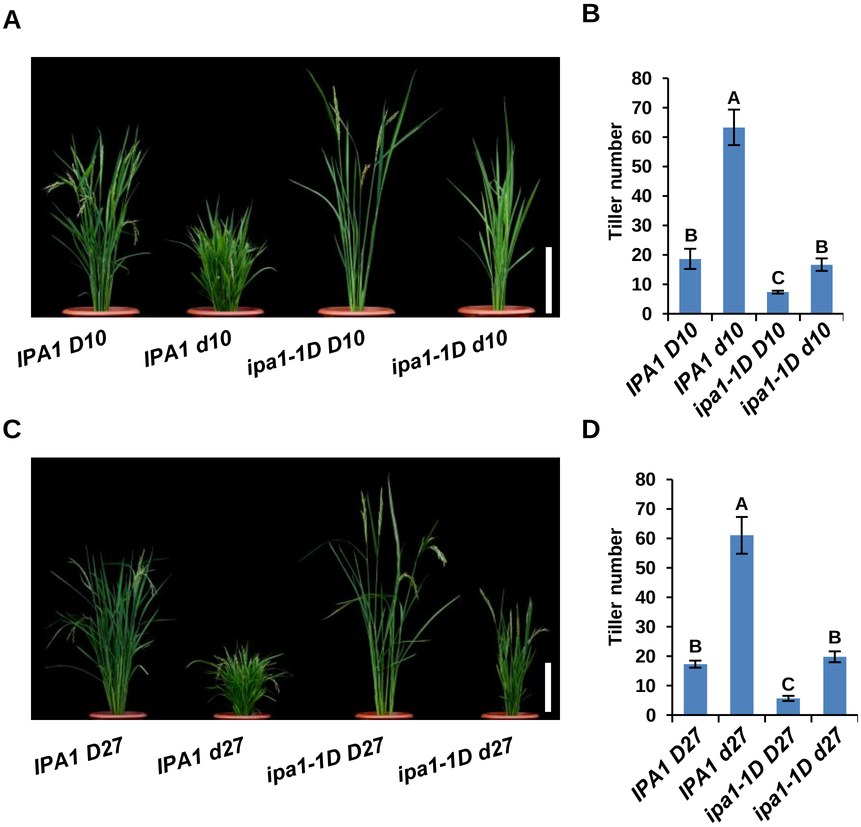

Figure S7 *ipa1-1D* represses the high tillering phenotype of *d10* and *d27*. **(A)** Gross morphologies of IPA1 D10, IPA1 *d10, ipa1-1D* D10, and *ipa1-1D d10*. Bar = 20 cm. **(B)** Statistical analysis of tiller number of **(A)**. Values are means ± SD (n = 8). Different letters at top of each column indicate a significant difference at $P < 0.05$ determined by Tukey's HSD test. **(C)** Gross morphologies of IPA1 D27, IPA1 *d27*, *ipa1-1D* D27, and *ipa1-1D d27*. Bar = 20 cm. **(D)** Statistical analysis of tiller number of **(C)**. Values are means ± SD (n = 8). Different letters at top of each column indicate a significant difference at $P < 0.05$ determined by Tukey's HSD test.
