# Supplementary material for: IPA1 functions as a downstream transcription factor repressed by D53 in strigolactone signaling in rice
Source: Cell Res. 2017 Aug 15;27(9):1128–41. doi: 10.1038/cr.2017.102 (PMC5587847; doi:10.1038/cr.2017.102)
Supplement: Supplementary information, Table S1 — Primers used in this study [file cr2017102x8.pdf]

Supplementary information, Table S1 Primers used in this study

Primer name	Sequence (5'-3')
Actin-F	CTTCATAGGAATGGAAGCTGCGGGTA
Actin-R	CGACCACCTTGATCTTCATGCTGCTA
D53 pro F	GAAGAAGCACTCACCAAACTCGT
D53 pro R	TGTACTCTGCAACTGCAACAGGA
Ubpro-qF	TCGGAGACCGTGCTAGGTTT
Ubpro-qR	GCCAGCGCCCATCGATT
IPA1-qF	TGCATTCCAAGGCTCCCCGC
IPA1-qR	TGCGGCAGCTGCGTTTTCCT
D53-qF	GAGGAGGATAGGAAACCTGTGCC
D53-qR	GTCTCCTTTCACTGCTGGTAC
GTAC-probe F	aaaaagcttctgttgcagttgcagcgtacagcagcagcatcagcaggatccttt
GTAC-probe R	aaaggatcctgctgatgctgctgctgtacgctgcaactgcaacagaagcttttt
D53-probe F	ttgctttccccctcctgttgcagttgcagagtacagcagcagcatcagcatcagcatat
D53-probe R	atatgctgatgctgatgctgctgctgtactctgcaactgcaacaggagggggaaagcaa
D53-probe-m F	ttgctttccccctcctgttgcagttgcagaatacagcagcagcatcagcatcagcatat
D53-probe-m R	atatgctgatgctgatgctgctgctgtattctgcaactgcaacaggagggggaaagcaa
IPA1-F	AAAactagtATGGAGATGGCCAGTGGAGGA
IPA1-N-R	AAAcccgggCCGCGGCGGCGGCGGCGGCGGCGGT
IPA1-SBP-F	AAAactagtatgTGCCAGGTGGAGGGGTGCGGCGCGGAT
IPA1-SBP-R	AAAcccgggAGGGGTTTGCGGCCTCCTCCGGCGCT
IPA1-C-F	AAAactagtatgTTGGCATCACGCTACGGTCGACT
IPA-R	AAAcccgggCAGAGACCAATCCATCGTGTTGCT
D53-F	AAAactagtATGCCCACTCCGGTGGCCGCCGCGA
D53-R	AAAcccgggACAATCTAGAATTATTCTTG
